# Supplementary material for: Are carbon emissions trading and green financial instruments synergistic? -Comprehensive quantitative research based on content analysis
Source: PLoS One. 2024 Mar 7;19(3):e0298601. doi: 10.1371/journal.pone.0298601 (PMC10919601; doi:10.1371/journal.pone.0298601)
Supplement: S1 Data — (ZIP) [file pone.0298601.s001.zip › Data/地级市Z_F工作报告.zip!/╔╜╬≈/╦╖╓▌╩╨/└┤╫╘╣1⁄2╓┌║┼í╛┬φ┐╦╩2╛▌═°í┐_2005.docx]

各位代表：

　　现在，我代表市人民政府向大会作政府工作报告，请予审议，并请市政协委员和其他列席人员提出意见。

　　一、2004年政府工作回顾

　　2004年，我们在中共朔州市委的领导下，按照市四届人大一次会议部署，坚持以“三个代表”重要思想和科学发展观为指导，团结带领全市人民，抓住机遇，争先发展，全市经济社会各项事业都取得了令人振奋的成就，成为建市以来经济发展最快、社会事业亮点最多、人民收入增加幅度最大、干部群众士气最高的一年。

　　宏观经济运行保持了前所未有的大好势头，各主要经济指标均创历史最好水平。综合实力快速增强，地区生产总值预计145．2亿元，增长工5.5％，分别高出全国、全省6．5个和2个百分点，人均突破1000美元小康标准，标志着一个新的发展阶段的到来。工业经济超常规发展，规模以上工业总产值增长39．3％，增加值增长26．4％，实现利税增长一倍以上，居全省前茅。财政收入保持了空前增长势头，总收入达到21．3亿元，增长44．72％，原定2007年实现第一个翻番时的目标已圆满实现；一般预算收入达到6．67亿元，增长26．26％。商贸经济更加繁荣活跃，社会消费品零售总额增长13．9％。城镇居民人均可支配收入达到7766元，增长10％。

　　“三农”问题受到格外重视，农业农村经济发展迈上新台阶。 2004年是近年来各级对农业农村经济发展支持力度最大、农民得到实惠最多的一年。农村税费改革扎实推进，农业税减免1个百分点的政策得到坚决贯彻，平鲁区实现了全部减免，全市共落实省转移支付及省市配套资金5099万元，农民人均负担由53．2元下降到 38元。支农投入进一步加大，兑现粮食直补资金1115万元，落实农业专项资金3045万元，极大调动了农民生产积极性。农业生产获得大丰收，粮食总产达到14．35亿斤，比历史最好的1996年增长 3．1％。生态畜牧经济区建设取得重大突破。大牲畜、生猪、羊饲养量均为历史最高年，其中奶牛达到12．6万头，增长15．7％。完成成片造林45万亩，其中退耕还林17．6万亩，累计退耕还林106万亩；种植牧草45万亩，累计达到120万亩。全市林草面积占到国土总面积的27．5％，全省首屈一指。农民收入较大幅度增加，人均纯收入预计2760元，净增240元，高出全省平均水平230多元。

　　招商引资工作取得明显成效，结构调整势头越来越好。签订引资引项合同117项，总投资187亿元，已开工项目33项，到位资金30．9亿元。结构调整无论铺开项目总数，还是投资总规模，都是历年来最多的。当年确定的25个市级重点调产项目，已建成17个，完成投资39亿元，其余8个今年上半年可全部建成。传统产业进一步发展壮大，新增煤炭生产能力800万吨、煤炭洗选能力1000万吨、电力装机容量60万千瓦。新兴产业成长步伐加快，乳品生产规模进一步扩大，以硅铝合金、硅锰合金为主的冶金业快速兴起，以高岭土、单晶莫来石和高标号水泥为主的新型建材业加速发展，一批新的特色旅游景点建设稳步推进。各级各部门抓调产、促发展的积极性越来越高，思路越来越宽，项目规模也越来越大，一个比学赶超的氛围已经形成，对我们今后发展的推动作用将是无法估量的。

　　基础设施建设进展顺利，发展环境进一步改善。公路建设取得新突破。平朔一级公路基本建成，右玉通市油路右玉至平鲁段建成，5条县际公路和11条通乡油路全部竣工，全市公路建设总投资达到5．76亿元，总里程1394公里，每万人拥有公路里程稳居全省第一。城市建设与管理水平进一步提高。城市总体规划上报国家建设部，县域城镇体系规划全面展开；安泰街建设顺利完成，污水处理厂、交通小区、电力小区已开工建设，垃圾处理厂国债资金计划已经下达。旧城改造步伐加快，东大街、马邑路、开发路中段等七条道路改造及开发取得重要进展。园林城市创建工作取得新成效，新增城市绿地面积29万平方米，城市绿化覆盖率由28％提高到 29％，人均公共绿地面积由13．5平方米增加到14平方米。软环境建设不断深入，以贯彻实施《行政许可法》为契机，进一步引深行政审批制度改革，积极推进政府职能转变，办事效率、服务水平均有所提高，政务公开经验在全国推广。

　　各项社会事业全面发展，精神文明和民主法制建设得到加强。建立健全了科技进步激励机制，出台了科学技术奖励办法，并设立了奖励基金。组织实施市级以上科技项目79项，其中国家级l项，省级24项，科技对经济增长的贡献率进一步提高。教育工作出现了三大新亮点：一是随着右玉“普九”验收的通过，全市“普九”任务圆满完成；二是高考成绩又有新突破，本科达线人数首次突破2500人，600分以上高分考生居全省前茅；三是高等教育开始起步，师范、农校专科阶段教学逐步展开。卫生事业得到较大发展。5个疾控机构项目基本完成，6个医疗救治项目基本完成4个，突发公共卫生事件应急指挥系统全面建立，特别是市中心医院的开工建设，标志着我市长期以来没有市级医院的历史即将结束。文化体育活动空前活跃，成功举办承办了“2l世纪中国文化产业论坛第三届年会山西论坛”会议、“山西省第三届广场文化艺术节”、“中国著名书画家朔州笔会”等一系列文化活动，人民群众精神生活更加丰富。计生工作进一步加强，获得省进步奖；环境达标成果得到巩固提升，城市空气质量二级以上天数全省第一。思想道德建设和学习宣传“三个代表”重要思想活动深入开展，群众性精神文明创建活动形式更加多样。 民主法制建设得到加强。市政府认真执行市人大及其常委会的决定和决议，自觉接受人大监督，积极听取市政协、各民主党派、人民团体及各界群众意见，办理人大代表意见、建议和政协委员提案196件。基层政权建设扎实推进。严打整治和综合治理工作逐步深入，社会秩序稳定。廉政建设不断加强，“三项治理”取得阶段性成果。土地、审计、宗教、人防、地震、气象、档案、老龄、残疾人事业等都取得了新成绩。

　　执政为民理念深入人心，事关人民群众生产生活的问题得到进一步解决。狠抓了八件实事的办理。解决了157个村、10．5万人的饮水困难，超计划2．5万人；完成了184所、5．9万平米的中小学危房改造，超计划46所；原定300个村、600公里通村油路任务，已完成304个村、1022公里；农村低保和农村新型合作医疗试点分别在平鲁和怀仁展开；农村沼气项目动工1000余户。加快了社会保障体系建设。各类保障资金达到3．5亿元。5993名国有企业下岗职工、14923名企业离退休人员按时足额领到基本生活保障金和养老费。新增就业岗位1．23万个，再就业人数达到5499人，城镇登记失业率控制在1．3％以内。高度重视社会救助工作，发放救灾款 810万元，对3332名五保老人实行了财政转移支付供给制，城镇4.76万名低保对象实现了应保尽保。继续推进扶贫攻坚，完成了58个村的搬迁任务，安置移民3186人；解决拖欠职工工资1．56亿元，其中农民工工资4698万元，分别占到拖欠总额的52％和81％；完成中低收入群体经济适用住房31．7万平米。人民群众生活水平进一步提高。

　　各位代表，过去一年的大好形势是有目共睹的，所取得的成就是历史少有的。这是市委坚强领导，市人大、市政协、各民主党派和人民团体鼎力支持，全市各级各部门和广大人民群众团结奋斗、不懈努力的结果。在此，我谨代表市人民政府，向为朔州改革开放和现代化建设作出重大贡献的全市人民致以崇高的敬意! 向所有关心、支持朔州改革发展大业的同志们、朋友们，表示衷心的感谢!

　　在肯定成绩的同时，我们也清醒地看到，在前进道路上，还存在着许多困难和问题。结构调整步伐仍显缓慢，新兴产业发展尤为不足，支柱产业单一化格局并未得到大的改观；农业综合生产能力尚未得到明显提高，农民增收基础并不稳固，增收渠道仍很狭窄；在有效解决弱势群体生产生活问题上，帮扶手段还很脆弱；社会事业发展相对滞后，就业压力、环境压力和安全生产压力依然很大，构建和谐社会的任务十分繁重；社会治安状况群众还不够满意，投资环境还不够宽松，干部队伍思想作风建设也有待进一步加强。对此，我们一定要居安思危，高度重视，下决心予以克服和解决。

　　二、2005年政府工作总的指导思想和主要目标任务

　　2005年是贯彻落实科学发展观、保持经济社会良好发展态势的关键一年，也是全面实现“十五”计划目标、衔接“十一五”发展的重要一年。新的一年，我们将面临更多难得的发展机遇。国际国内宏观经济形势持续向好，能源需求更加旺盛，主要工业品价格将继续保持高位运行，使我市经济增长有了充分的市场支撑；全球性结构调整和产业转移步伐进一步加快，国家对煤、电、油、运等产业和农业的扶持力度进一步加大，为我市结构调整拓展了新空间，增添了新力量；我市连续几年经济持续快速增长，社会全面进步，特别是经过数年来坚持不懈的结构调整，培育了一大批新的增长点，经济发展潜力巨大，为我们更快更好地发展奠定了坚实的基础。可以预见，新的一年必将又是形势喜人的一年。我们有信心、也有决心在市委的坚强领导下，团结带领全市人民，努力夺取经济社会发展的更大胜利！

　　按照市委三届十次全会“抓住一个根本(提高党的执政能力)，争做两个排头兵(全省新型能源基地和全省生态畜牧基地建设排头兵)，推进城市化进程，到2007年再造一个新朔州”的总体要求，今年政府工作总的指导思想是：以邓小平理论和“三个代表”重要思想为指导，认真贯彻党的十六大和十六届三中、四中全会精神，更好利用重要战略机遇期，全面落实科学发展观，以执政为民谋发展为主题，紧紧围绕加速经济发展、构建和谐社会、提高人民生活水平三大目标，突出经济结构调整、解决“三农”问题、加快城市化进程、推进科教兴市四个重点，抓住扩大社会就业、健全保障体系、维护群众切身利益、加强资源环境保护四个热点，优化发展环境，扩大招商引资，加强民主法制建设，推进政府职能转变，实现全市经济社会跨越式发展，为建设富强朔州、生态朔州、人文朔州、信用朔州、和谐朔州而奋斗！

　　落实这一指导思想，最根本的就是要坚持以科学发展观来统领经济社会发展全局。这是我们全面建设小康社会、积极推进社会主义现代化建设必须始终遵循的一条基本准则。能源工业城市的基本经济特征和建市时间较短、各项社会事业发展欠帐较多的基本市情，决定了我们在贯彻落实科学发展观问题上比其他任何地方都更加紧迫、更加突出。我们一定要从有效消除当前经济运行中的不健康不稳定因素、差力化解经济社会发展中的各种深层次矛盾、确保经济社会全面协调可持续发展的大局出发，严格按照科学发展观的要求来谋划和展开新一年的工作。要以解决“四个不协调”问题为重点，即经济持续健康发展与单一产业构成上的不协调、经济发展与社会进步之间的不协调、城乡经济社会发展及居民生活水平上的不协调、经济建设与资源环境保护的不协调，努力把握好四条原则：一是坚持以提高经济增长的质量为核心，高度重视经济结构的优化升级、经济增长方式的转变和科技进步，努力实现速度、结构、质量和效益的统一。二是坚持全面协调发展，物质文明、政治文明和精神文明一起抓，高度重视科技、教育、文化、卫生等事业，特别是农村教育、卫生等事业发展，在确保经济快速增长的同时，努力促进社会的全面进步，实现经济社会的和谐协调。三是坚持可持续发展，在开发利用自然中，高度重视资源节约与环境保护，加快节约型社会建设，努力实现人与自然的和谐相处。四是坚持以人为本，高度重视维护和实现广大人民群众的利益，努力促进人的全面发展，最终形成当前与长远、经济与社会、人与自然、城乡之间、区域之间高度和谐的社会。2005年全市经济社会发展的主要预期目标初步确定为：地区生产总值170亿元，增长13．5％；财政总收入26．06亿元，增长 22．35％；一般预算收入7．85亿元，增长17．73％；全部工业增加值 89亿元，增长22％；粮食总产稳定在13亿斤以上，全社会固定资产投资72亿元，增长20％；社会消费品零售总额51亿元，增长 13．7％；居民消费价格指数控制在103％左右；海关进出口总额 2200万美元，增长12％；城镇居民人均可支配收入8500元，增长 10％；农民人均纯收入3000元，增长8．7％；人口自然增长率控制在千分之八以内。

　　为实现上述奋斗目标，要着力抓好七个方面的工作：

　　(一)加快新型能源基地建设，加速新兴产业发展，推动经济结构优化升级

　　加快结构调整，转变增长方式，提高经济增长的质量和效益，是一项长期而紧迫的重大战略任务，必须坚持不懈地抓下去。

　　要紧紧抓住加快新型能源基地建设、加速新兴产业发展这一核心内容。能源工业是立市之基，发展之本，没有能源工业的做大做强，我们的发展就将失去最主要的支柱；但新时期的能源工业发展，必须走新型化的道路，没有能源工业的新型化，就将失去能源工业长盛不衰的生命力。要改变单纯把煤炭工业实力定位于量、定位于“大”的思想，以提高产业素质、提高综合竞争能力、提高经济效益、提高可持续发展能力为核心，以规模化、洁净化、增值化、现代化为方向，按照“统筹规划、优化布局，资源整合、联营改造，延伸加工、限量增效，安全生产、持续发展”的方针，加快煤炭产业新型化进程。大力提高产业集中度。加大资源整合力度，推进煤炭资源向大企业大集团集中，形成一批集产、运、销和加工于一体的企业集团；加快现有煤矿技术改造。实施高产高效矿井改扩建工程，重点发展百万吨以上矿井，年内力争完成30座矿井改造，新增生产能力600万吨；加快一批大型矿井建设，坚决关闭9万吨以下小煤矿，逐步淘汰不具备改造条件的30万吨以下煤矿。大力提高产业技术水平。加快采煤方法改革，力争用两年左右时间使全市所有煤矿全部实现综合开采或壁式开采，促进煤炭企业机械化水平、单井生产能力、安全保障能力和资源回收率大幅提升。坚持走转化增值的路子，不断提升洗选加工能力。较大型煤矿和矿区都要配套建设洗选设施，提高煤炭质量和市场竞争力；大力发展煤炭深加工，积极推进煤转油、煤制甲醇等清洁燃料的开发利用，延伸产业链，提高附加值。要加强管理，堵塞漏洞，不断提高煤炭行业经济效益，使资源优势真正转化为经济优势。要通过多种措施，尽快把我市建设成为全国最大、全国领先的新型煤炭基地。电力工业要抓住机遇，上新上大、积极与大企业大集团联合，加快推进脱硫除尘、空气冷却大型发电机组和中煤煤矸石电厂建设，确保大唐2*50万千瓦、平朔2*5万千瓦机组如期投产，全力推进平朔2*30万千瓦、王坪 2*20万千瓦、朔能2*20万千瓦、永皓2*5万千瓦、应县2*5万千瓦、刘家口2*13.5万千瓦电厂建设，力争年内全市电力装机容量达到360万千瓦，2006年达到500万千瓦，使我市成为位居全国前列的新型火电基地。高度重视新兴产业发展，努力构建多元化产业体系，加快资源型城市转型步伐，拓宽发展空间，增强抵御和化解市场风险的能力。突出抓好以煤制甲醇为主的新型化工业、以硅铝合金为主的金属和以高岭土为主的非金属新型建材业、以中高档日用瓷为主的新型陶瓷业和特色生态旅游业四大产业。煤制甲醇是煤炭加工转化的主要方向，具有极其广阔的发展前景，要抓好正在筹备的4个项目，争取神华煤转油项目在我市落户，实现煤化工业的快速起步。把我市建设成为全省乃至全国重要的清洁燃料供给基地。新型建材业在我市发展势头良好，特别是硅铝合金、硅锰合金和铁合金，发展步伐很快，要积极推进晋能“双10万吨”硅铝合金项目建设，并依托丰富的煤电和铝矾土资源优势，争取再上几个较大规模的项目，并加快下游产品开发，力求走出一条从无到有、一步到强的冶金业发展路子，形成煤、电、铝产业链条。陶瓷业要增加品种、提高档次，尽快由低档产品向中高档产品迈进，由日用瓷向建筑瓷、工艺瓷、工业瓷迈进。生态旅游业要按照“科学规划、市场运作、多元投入、打造品牌”的方针，围绕大运高速公路黄金旅游带，加快应县木塔、朔城区崇福寺、怀仁金沙滩和清凉山、右玉杀虎口、山阴广武城等一批特色景点景区的建设，扩大旅游业规模。同时，要重视高新技术产业发展，重视运用高新技术对现有产业进行改造，努力提高产业技术含量。要按照“减量化、再利用、资源化”原则，以推进资源节约、综合利用、清洁生产为重点，大力推进循环经济发展，构建资源节约型经济增长模式。

　　要牢牢把握好产业规划、项目建设、园区化发展等重要环节。要引导各级各部门，着眼长远，着眼全局，尊重科学，尊重规律，认真把握好主导产业确立、发展目标定位、项目选择、区域布局几个重点，充分考虑国家的产业政策、市场前景、资源状况和产业基础，切实做好各自的总体规划，增强可行性，避免盲目性。对已经确立的主导产业，要逐一加以研究，对每个产业的详细发展规划、具体政策、重点项目和推进措施，都要进一步明确并加以完善。要坚持项目带动，在大上项目的基础上，突出上大项目，力求尽快抓出一批突破性和带动性较强的重大项目。认真做好项目前期工作，为引资引项奠定良好基础；健全项目建设目标责任制，加强对项目建设的指导与服务；不断创新资金筹措机制，用好政府调产基金，建立有效的投入和退出机制，加大对项目建设的支持力度，吸引金融资金和社会资金，确保项目建设的顺利进行。今年初步确定了30个重点项目，总投资154亿元，其中平朔1000万吨井工矿、华美奥兴陶500万吨煤矿、大唐2X50万千瓦机组等13个项目要力争投产，有6个项目要部分投产，3个项目要基本建成。进一步加快园区建设。园区化发展的方向不能变，加快园区建设的力度不能减。对已经确定的各个园区，特别是晋能冶金工业园区、刘家口煤电工业园区、木瓜界煤电工业园区、红旗牧场科技园区等重点园区，要一个一个地完善规划，明确任务，确定专人负责，加快基础设施建设，做好企业入园区工作，力争年内有一个大的发展。要认真处理好园区建设与耕地保护的关系，严格依法办事，严格审批程序，用好已征用土地，用好废弃农田和非基本农田，盘活观有土地存量，并坚持占补平衡，加大复垦开发力度，保证园区建设用地。

　　要紧紧依靠扩大招商引资、实施大企业大集团战略和加快非公有制经济发展来推动。坚持把招商引资作为保障经济投入、推进结构调整的最大动力和最主要手段，不断扩大招商引资成果。要加大政府推动力度，进一步健全和完善有关领导体系、目标责任、奖惩办法，确保招商引资深入持久地开展下去；要靠资源吸引，充分发挥资源优势，加强政策引导，凡投资能源工业，特别是煤炭采掘业者，要求其配套建设相关延伸加工项目和其他地面企业，以资源换资金，以资源换项目，走出以地下促地上、以传统产业衍生带动新兴产业发展的路子。认真实施大企业大集团战略，充分发挥大企业大集团在结构调整中的骨干带动作用，进一步加快大企业大集团的集聚，支持现有大企业大集团发展，积极培育扶持地方企业成长壮大，通过企业做大做强，带起优势园区，形成优势行业、优势产业和强势产业基地。放手放胆发展非公有制经济，全面放宽市场准入和投资领域，拓宽非公有制经济发展空间；建立有效帮扶体系，加快信用担保体系建设，促进非公有制经济更快发展；支持鼓励非公有制企业承担重大调产项目，发挥其在调产中的生力军作用。

　　(二)加快生态畜牧基地建设，加大对“三农”的支持保护力度，提高农业和农村经济发展水平

　　坚持把“三农”问题作为重中之重，格外关心农业，关注农村，关爱农民，进一步加大对“三农”的支持保护力度，紧紧抓住生态畜牧基地建设这个龙头，带动农业农村经济结构战略性调整，实现农业农村经济发展水平的整体提高和农民收入的有效增加。

　　以科技手段为支撑，提高生态畜牧基地建设水平。改变传统饲养方式、饲食结构、繁育技术和防疫手段，推动畜牧业向高科技、现代化方向迈进。大力推进饲草饲料革命。抓好10万亩带穗青贮玉米种植，带动全市150万亩优质高蛋白饲用玉米基地建设；坚持立草为业，新增 10万亩多年生优质牧草，形成粮、经、草三元格局。推进良种革命，建设市县两级奶牛配种服务中心，实现成年母牛统一供精冷配。加快畜牧园区建设，扩大养殖规模，提升养殖质量。突出抓好一批标准化养殖园区建设，带动畜牧业超常规发展，全市奶牛力争达到15万头。加快龙头企业发展，提升产业化水平。继续扶持建设一批饲草加工企业，提高牧草利用率；抓好雁门乳业15万吨、康喜10万吨、春城5万吨三个液态奶项目及应县玉米淀粉、朔城区高芽糖、山阴燕麦加工、平鲁无矾粉丝、右玉真空冷冻干燥食品等项目，带动农副产品加工业上水平，力争全市农副产品加工转化率达到50%以上。各级政府要切实加大科技推广力度。抓科研，建立市县两级科研中心、增强科技攻关能力；抓服务，加强防疫体系建设，搞好市畜牧兽医服务中心完善和6个县区实验室、30个乡镇中心站建设；抓示范，典型引路，特别注意培养在标准化园区建设、饲养方式变革上的典型；抓培训，开展“50万农民科技大培训”活动，鼓励有条件者走出去学习；抓队伍，加强同科研院所合作，引进高科技人才，鼓励大中专学生回乡创业；抓投入，对优质饲料玉米种植、饲草机械化收割、青贮窖建设、标准化园区建设给予补贴。坚持生态效益与经济效益并重，积极推进生态建设。完成工程造林70万亩，发展灌木林50万亩，经济林5万亩；高标准完成平朔一级公路、世纪大道、右玉通市油珞通道绿化工程；完善大运高速公路和109国道绿化带工程。

　　进一步加大对“三农”的扶持保护力度，激发农业农村经济发展活力。坚持“多予、少取、放活”的方针，各项支农措施的力度只能加大不能减弱，已经给农民的实惠只能增加不能减少，并且要随着财力的增强，逐步加大支农力度。继续执行严格的耕地保护政策，稳定耕地面积，稳定粮食生产，保护农民土地权益。对失地农民的补偿安置要由一次性经济补偿为主转向以建立养老保险为主。继续贯彻执行去年出台的有关支持农业农村经济发展的八项政策，认真落实有关粮食直补政策。积极调整国民收入政策和财政支出结构，建立支农资金稳定增长机制，确保支农资金增长不低于当年财政收入增长；继续深化农村税费改革，年内全部减免农业税，大幅度减轻农民负担。推进粮食流通体制改革，严格执行最低收购价制度；推进农村金融体制改革，加大对“三农”的信贷支持力度；大力发展农村中介服务组织，为农民更好进入市场铺路搭桥。

　　加快农村基础建设和各项事业发展，增强农业农村经济发展的内在实力。继续抓好京津风沙源治理、首都水资源可持续利用和世行贷款盐碱地治理工作，新造耕地2万亩，建设高标准节水园区4万亩。继续解决好人畜吃水问题，力争年内解决7．2万人的饮水困难，完成2．5万人的氟改水工程。进一步加快农村小康建设，力争2个乡镇、29个村、1．4万人基本达小康，4个乡镇、162个村、13万人步入宽裕型小康行列。继续推进移民并村工作，扶贫移民1．31万人，生态移民1200人。

　　(三)加快城市化进程，增强城镇辐射带动力，促进城乡统筹发展

　　坚持以工促农、以城带乡，积极加快城市发展步伐，构建有利于城乡融合的体制机制，增强城镇对乡村的辐射带动力，改变城乡二元结构，实现城乡共生共长、统筹发展。

　　加快城镇基础设施建设，提高城镇承载能力。进一步扩大城市路、电、水、气等基础设施容量，完成平朔一级公路扫尾工程，抓好南环路、顺义路、开发北延路等城市道路的建设与改造，加快市体育馆、污水处理厂、垃圾处理厂、七里河改造、市委西区、电力小区、医疗卫生中心、农牧科技中心、教育园区、旧城改造等重点工程建设，完善城市功能。加快城市绿化步伐，新增绿化面积31.7万平方米，新增水面5万平方米，创建省级园林城市。加强城市管理。完成城市部分区域、街道的控制性规划和详细规划，加强和规范社区建设；下决心整治脏乱差问题，切实加大对烟尘、污水的治理力度，推进洁净城市、文明城市建设。城市空气质量二级以上天数要达到200天以上，污水处理厂力争年内完工，市区污水处理率达到80%左右。加快以县城为主的小城镇建设。完成县域城镇体系规划，加快县城基础设施配套和旧城改造，启动县区住房公积金制度，加快中低收入群体经济适用住房建设。积极推进其他建制镇建设。继续加大公路建设力度，建设5条一级公路(南环路、西环路、怀仁城东过境公路、高速公路山阴连接线和广武连接线)，改建 7条二级公路(元元公路、苑家辛庄至偏岭、高速公路毛皂连接线、朔广线朔州至化庄、平万线、右玉通市油路续建、朔洗线应县县城段改线)和5条通乡油路(山阴马营乡和古城镇、应县义井乡、朔城区张蔡庄乡、怀仁毛皂镇)，完成200个村通油路任务，新建3座桥梁(怀河线桑干河大桥、朔广线南邢家河大桥、应县西朱庄大桥)，新建和改建20个乡镇级汽车站。

　　加快城镇经济发展，增强城镇国辐射带动力。城市带动力，主要是经济带动力。要特别重视市级经济发展，积极引进和兴建几个大的调产项目，千方百计盘活做大现有企业，大力发展城市服务业和流通业，充实市级经济实力。积极发展县域经济。紧紧依托县城和集镇这一农村城镇化的前沿，合理布局生产力，努力把县城和大的集镇建设成为具有生产、贸易、交通、居住、消费等多种功能的县域经济增长中心。要突出特色，努力挖掘自身发展优势，因地制宜，分类指导，确定各具特色的主导产业，实行重点开发，在资源优势中培育地方特色，在传统产品中筛选优势品牌，打造特色产业和名牌产品。要把非公有制经济作为发展县域经济的主体力量，激发城镇居民和农民的创业热情，促进非公有制经济的快速成长，努力增强县级财政自主增长的能力。

　　要创造各种有利条件，加快农村剩余劳动力向城镇转移。城镇化进程，就是农民进城并扎根立业的过程。要把扩大农民就业、促进农村剩余劳动力转移作为市县经济发展，特别是县域经济发展的重要任务，在产业选择、区域布局上充分考虑农民就业问题，努力增加农民就业岗位。加大对进城农民自主创业的扶持，对进城从事小型加工业、商贸服务业的农民，在小额贷款上与从事农牧业同等待遇。要把农村劳动力就业培训作为一件大事来抓，不断提高农民工素质。推进户籍制度改革，放宽农民进城就业和定居条件；把在城市有固定工作和住所的进城农民视同常住人口，在住房、就学、社会保障等方面逐步走向平等。要创造良好环境，既注重降低企业的商务成本，又注重降低农民工的生活成本，使农民既能进得来、有事干，又能留得下、住得起。

　　(四)落实国家宏观调控政策，强化财税金融工作，增强政府宏观调控能力

　　认真落实、自觉顺应国家宏观调控政策，保持宏观经济健康运行。坚决贯彻落实国家宏观调控政策，严把土地征用、项目审批等关口，抑制部分行业的过度投资和盲目发展，限制低水平重复建设和高污染项目实施。认真研究国家产业政策，瞄准国家扶持的产业和领域，积极做好项目申报和资金争取工作。加强对经济运行的协调、监测、监控。认真搞好经济普查，科学编制“十一五”规划，合理运用经济、法律和必要的行政手段，做好本地区煤、电、油、运调度，增力口有效供给，及时消除经济运行中的不稳定因素。

　　继续做好财税工作，确保财政增收。深化部门预算、国库集中收付、政府采购和收支两条线管理制度改革，完善转移支付制度和出口退税机制。坚持依法治税，强化征管，并根据市场价格等因素的变化，实行动态管理，做到应收尽收。注意地方财力的增加，加强预算外资金、非税收入和非经营性资产管理。严格执行国务院《财政违法行为处罚处分条例》和省委、省政府关于深化预算管理改革的意见，加强预算执行审计和财政决算审计，强化预算管理，继续执行一般性支出零增长政策，严格控制财政供养人员增长，依法保证重点支出，确保收支基本平衡。

　　切实改进金融服务，支持地方经济发展。在信贷适度从紧的情况下，金融部门要积极寻求有效途径，切实改进对中小企业的信贷评估、审批和发放制度，努力增加信贷规模，改善金融服务。地方要支持金融部门加强监管，防范和化解金融风险。稳步推进农村信用社改革。

　　规范市场经济秩序，加快信用体系建设。坚持标本兼治，完善市场监管体系，形成长效监督机制，维护正常的市场秩序。重点抓好直接关系人民群众安危健康的食品、药品等专项整治，严厉打击制假售假、商业欺诈等不法行为。加快信用朔州建设，增强全社会的信用意识，完善激励守信、惩戒失信的社会信用监督约束机制。建设和完善市县两级企业信用信息网络平台。

　　(五)大力实施科教兴市战略，积极发展各项社会事业，实现全面协调发展

　　要把教育放在优先发展的战略地位，按照“巩固充实农村教育、加强完善城市教育、谋划发展高等教育”的思路，推进各类教育共同发展。进一步加快城市基础教育设施建设，着力解决入学难和班容量大的问题。加强普通高中教育，完成l一2所省级和3—4所市级示范高中创建任务，提高教学质量。大力实施“农村教育振兴工程”，基本完成中小学危房改造，充实农村师资队伍，合理调整学校布局，巩固建设一批高标准农村寄宿制学校，提升“普九”成果。深化教育改革，积极推进校长聘任制和教师招聘制。继续加快职业教育、成人教育发展。整合农校、师范、技校、电大资源，谋划建设一所规模较大的高等职业技术学院。力争用5年左右时间，建成城乡教育协调发展、基础教育与高等教育统筹发展、义务教育均衡发展的现代教育体系。不断提高科技发展水平。以提高科技创新能力为核心，加快高标准科技创新示范基地建设；支持、引导有条件的企业建立技术研发中心，增强企业自主创新能力；扶持发展中介科技服务机构，加快民营科技企业发展；广泛开展科技交流与合作，加快适用新技术、新成果的推广应用。开通全市科技信息网，建立科技项目库、成果库、专家人才库、企业商务信息数据库，实现资源共享。大力发展卫生事业。继续推进新型农村合作医疗试点工作，加强乡镇卫生院建设，切实解决好一些农村因贫致病、因病致贫的问题。健全和完善疾病防控体系、突发公共卫生事件救治体系、重大疫情信息网络体系和卫生执法体系，抓好市疾控中心的启动运营，完成市急救中心和中心医院建设任务，加大医疗卫生市场整顿力度。实施人才强市战略，紧紧抓住培养、吸引、用好三个环节，不断完善选人用人、利益激励、人才流动三大机制，加强党政人才、企业经营管理人才和专业技术人才三支队伍建设，为全市各项事业发展提供智力支持。加强城乡文化、体育设施建设和广播电视网络建设，推进文化市场净化工程，办好首届全民运动会，繁荣和活跃群众文化体育生活。强化人口和计划生育工作，稳定低生育水平。加强环境保护，开展环保城市创建活动，大力实施蓝天、碧水工程，推进固体废资源化利用及安全处置、 工业污染源全面达标、取缔关停五小企业等工作，力争3—5年内建成全国环保城市。发展档案、史志、气象、测绘、防震减灾等事业。关心支持老龄、妇女、儿童、残疾人、民族宗教等工作。加强国防教育和民兵预备役部队建设。加强社会主义精神文明建设和公民道德建设，深入开展文明单位、文明村镇、文明社区等群众性创建活动。

　　(六)坚持以人为本、执政为民，维护群众切身利益，着力构建和谐社会

　　政府是人民的政府。人民政府就必须以人民的利益为根本。要坚持把为人民群众谋利益作为政府一切工作的出发点和落脚点，时刻把人民群众的冷暖记心头，从最基层人民群众的安危健康想起，从满足最基本的衣食温饱需求做起，自觉运用人民赋予的权力，积极为人民群众办实事、办好事，帮民富、解民忧、保民安、化民怨，促进社会公平，维护社会正义，构建和谐社会。

　　千方百计扩大就业。就业乃民生之本。要把扩大就业放到突出位置，全面落实就业再就业目标责任制和促进就业的各项政策措施，大力开发就业岗位，重视发展劳动密集型企业、第三产业特别是社区服务业，力争新增就业岗位1．5万个。继续实施再就业援助制度，市政府要拿出2000万元专门用于再就业援助，公益岗位要优先安置“4050”等就业困难人员。建立和完善公共就业服务体系，加强街道社区劳动保障平台建设，做好就业指导和培训工作，促进下岗人员多形式就业。统筹做好高校毕业生就业、复转军人安置、城镇新增劳动力和农村富余劳动力就业工作。

　　加快完善社会保障和救助体系。确保企业离退休人员养老金按时足额发放，进一步扩大养老保险覆盖面，形成只要开办企业、招用职工就要参保的机制。积极探索机关事业单位养老保险制度改革，完善机关和企事业单位人才流动中的社会保险衔接办法。扩大失业和生育保险范围，推动事业单位和非公有制企业参加失业保险。完善医疗保险制度，扩大工伤保险覆盖面，稳步推进农村社保工作。加大劳动保障监察执法力度，企业用工必须签订劳动合同。抓紧建立以最低生活保障和灾民救助制度为基础，以医疗、教育、住房等专项救助和社会互助为补充，以优惠政策相配套的覆盖城乡的社会救助体系。

　　努力增加城乡居民收入。搞好农民外出务工的组织引导工作。加大对拖欠农民工工资问题的检查和处罚力度，力争年内基本清理完毕并不再发生新的拖欠。认真检查职工最低工资制度执行情况，督促企业按时足额发放职工工资。各级财政要首先保证行政事业单位人员工资发放，并积极落实中央和省有关增资政策，不断增加公职人员收入。进一步改革机关事业单位工资制度，完善公务员职级工资制度，推进国家公职人员福利待遇货币化。

　　切实维护社会稳定。完善安全生产管理体制和机制，增加安全生产投入、消除各种隐患。特别要加大煤矿安全生产专项整治和执法监察力度，坚决杜绝重特大事故发生。重视处理好农村征地、城镇房屋拆迁、国有企业改制等涉及群众利益的问题。加强信访工作，依法、及时、合理地处理好群众反映的问题。推进社会治安综合治理，严厉打击各种刑事犯罪，特别是黑恶势力犯罪、严重暴力犯罪，维护正常社会秩序，确保人民群众生命财产安全。

　　要继续为群众办好八件实事：一是全部减免农业税和农业税附加；二是解决160个村、7．2万人的吃水困难；三是完成18．7万平方来中小学危房改造，全市基本完成中小学危房改造任务；四是对1．2万名生活无保障的农村残疾人家庭成员全部实行低保；五是完成200个村、800公里村通油(水泥)路任务，基本实现“两个村村通”(村村通油路、村村通客车)、“两个1小时通达”(市到县区、县区到乡村)；六是市区范围内建设一所小学、一所初中，缓解入学难和班容量大的问题；七是市县两级共同出资，为每个县区购置2辆直接服务于农村的医疗卫生巡回救治车；八是构建贯通全市城乡，集视频、数据、语音于一体的有线广播电视综合传输网，为广大群众提供更多高质量电视节目和更便捷的信息服务。

　　(七)加强政府自身建设，提高执政水平，优化发展环境

　　努力建设一个忧民所忧、乐民所乐的服务政府，一个奋发有为、勇于任事的责任政府，一个依法行政、公正严明的法治政府，一个公开透明、廉洁自律的清明政府，一个勤政务实、协调运转的高效政府，以良好的形象凝聚群众，以模范的行动感染群众，以扎实的工作带动群众。

　　要科学执政。以建设学习型政府为重点，坚持用最新理论和最新知识武装干部，不断增强工作的主动性、自觉性和预见性，提高驾驭市场经济和管理社会的能力。以建设服务型政府为重点，积极推进政府职能转变，真正把政府职能转到经济调节、市场监督、社会管理和公共事务上来，转到创造良好发展环境上来，更好适应市场经济需求，集中力量办大事。积极推进政府决策的科学化、民主化进程，建立和完善重大问题集体决策制度、专家咨询制度、决策评估制度、社会公示和听证制度、决策责任追究制度，使我们的决策更加符合朔州的实际，更加符合人民的利益。

　　要依法行政。要依法治“官”，依法治“权”，依法治事。各级政府机关要带头遵法守法，严格依法行政，依法办事。加快建立权责明确、行为规范、监督有效、保障有力的行政执法体制，减少执法层次，规范执法行为，着力解决多头执法、重复执法和交叉执法问题，推进文明执法、公正执法。推行行政执法责任制和错案追究制，坚决查处徇私枉法、执法犯法、欺压百姓等违法行为。建立健全有效的行政监督体制和机制。各级政府要自觉接受同级人大及其常委会的监督，认真执行人大决议，定期向人大报告工作；积极接受人民政协的民主监督，认真听取各民主党派、无党派人士和各人民团体的意见；加强基层民主建设，继续推进“三务”公开，强化基层民主监督；拓宽新闻监督、公众监督渠道，做到有权必有责、用权受监督、违法受追究，着力解决权力与利益挂钩、与责任脱钩的问题，确保人民赋予的权力真正服务于人民。

　　要从严治政。全面落实党风廉政建设责任制，教育各级干部要常修为民之道，常思贪欲之害，常怀律己之心，严格自律，警钟长鸣。继续推进“三项治理”工作，严格执行领导干部经济责任审计和重点资金、重点项目、重点工程审计，健全土地出让、建设项目招投标、资产交易和政府采购制度，从源头上预防腐败。坚决查处违法违纪案件，毫不手软地清除公务员队伍中的腐败分子。坚决整治乱摊派、乱罚款、乱收费、乱检查等行业歪风。要反对“乱作为”，更反对“不作为”。切实加强对县区政府和所属部门的管理约束，确保政令畅通，杜绝推诿扯皮，防止政府权力部门化、部门权力个人化。建立严格的目标责任制，努力使每个政府干部都能谋事干事，做到人人有事干，事事有人干。强化督促检查，以事查人，以人查事，政绩公开。要赏罚严明，整治“当官不负责任、掌权不受约束、只吃俸禄不干实事”的问题，抓出谋大事、干实事的冲天干劲，抓出敢于负责、勇于开拓的气魄。

　　要务实施政。大力弘扬实事求是、求真务实的精神，努力营造说实话、道实情、办实事、求实效的良好氛围，狠抓落实，苦干实干，确保各项目标圆满实现。牢固树立科学发展观，既看显绩，也重潜绩，使我们的工作真正经得起历史的检验。树立正确的政绩观，坚决杜绝劳民伤财的“形象工程”、“观光工程”。积极转变工作作风，改进服务态度，提高服务质量，牢固树立环境就是资源、环境就是效益、环境就是发展资本、发展空间的观念，实现“政府创造环境、人民创造财富”。

　　各位代表，朔州是一个极富生机与活力的地方，朔州的明天必将是无比光明灿烂的!现在，我们已经站到了一个新的历史起点上，让我们在中共朔州市委的坚强领导下，高举邓小平理论伟大旗帜，坚持以“三个代表”重要思想和科学发展观为指导，紧紧团结和依靠全市人民，抓住机遇，奋勇争先，为实现全市经济社会的跨越式发展而努力奋斗!
